# Morphological and Genetic Characterization of Maize Landraces Adapted to Marginal Hills in North-West Italy

**DOI:** 10.3390/plants13071030

**Published:** 2024-04-05

**Authors:** Giovanni Maria Di Pasquale, Lorenzo Stagnati, Alessandra Lezzi, Alessandra Lanubile, Adriano Marocco, Graziano Rossi, Matteo Busconi

**Affiliations:** 1 Department of Sustainable Crop Production, Università Cattolica del Sacro Cuore, Via Emilia Parmense 84, 29122 Piacenza, Italy; 2 Research Centre for Biodiversity and Ancient DNA, Università Cattolica del Sacro Cuore, Via Emilia Parmense 84, 29122 Piacenza, Italy; 3 Department of Earth and Environmental Sciences, Università di Pavia, Via S. Epifanio 14, 27100 Pavia, Italy

**Keywords:** maize landraces, SSR, morphological characterization, genetic characterization, agrobiodiversity, traditional germplasm

## Abstract

The growing interest in maize landraces over the past two decades has led to the need to characterize the Italian maize germplasm. In Italy, hundreds of maize landraces have been developed, but only a few of them have been genetically characterized, and even fewer are currently employed in agriculture or for breeding purposes. In the present study, 13 maize landraces of the west Emilia-Romagna region were morphologically and genetically characterized. These accessions were sampled in 1954 from three provinces, Modena, Parma, and Piacenza, during the characterization project of Italian maize landraces. The morphological characterization of these 13 accessions was performed according to the UPOV protocol CPVO/TP2/3, examining 34 phenotypic traits. A total of 820 individuals were genotyped with 10 SSR markers. The genetic characterization revealed 74 different alleles, a F_ST_ mean value of 0.13, and a Nm mean of 1.73 over all loci. Moreover, AMOVA analysis disclosed a low degree of differentiation among accessions, with only 13% of genetic variability found between populations, supporting PCoA analysis results, where the first two coordinates explained only 16% of variability. Structure analysis, supported by PCoA, showed that only four accessions were clearly distinguished for both K = 4 and 6. Italian landraces can be useful resources to be employed in maize breeding programs for the development of new varieties, adapted to different environmental conditions, in order to increase crop resilience and expand the maize cultivation area.

## 1. Introduction

Maize (*Zea mays* ssp. *mays* L.) is one of the most important crops in Italian agriculture and worldwide. Domesticated around 9000 years ago from teosinte (*Zea mays* ssp. *parviglumis* [1]) in Central America, maize was initially imported into Europe by the first shipments of Cristoforo Colombo [2]. However, it took about a century of attempts on different genotypes to adapt to the new environments, gain importance as an agricultural crop, and spread throughout the Italian territory.

Over the last four centuries, farmers applied empirical selection to adapt maize to different environments and farmers’ needs, developing numerous landraces to the point that the Mediterranean Basin can be considered a secondary center of maize differentiation [2]. At that time, farmers maintained landraces as open-pollinated populations and sometimes hybridized with other maize cultivars from continuous exchange and trade, leading to the formation of populations with highly heterozygous and heterogeneous plants [3,4].

Since the end of the Second World War, the Italian agricultural scenario has changed profoundly due to the introduction of maize dent hybrids from the USA, leading to the disappearance of most of the Italian maize agrobiodiversity [5]. To limit the loss of this important source of germplasm, in 1954 an intensive collection of the main traditional maize cultivars was carried out on the entire national territory by the “Stazione Sperimentale di Maiscoltura” of Bergamo. This sampling resulted in 562 different accessions from 65 provinces, classified into nine racial complexes, 34 landraces, 65 agroecotypes by Brandolini and Brandolini [2,5], which are maintained by the CREA-Cereal and Industrial Crops (CREA-CI) in Bergamo.

In the last two decades, breeders and farmers have rediscovered the importance of landraces and crop wild relatives as an essential source of genetic material. Landrace populations, with their high level of genetic variability and fitness to natural and anthropological environments, provide a valuable source for potentially useful traits and an irreplaceable bank of co-adapted genotypes [6,7]. Breeders may harness useful genes and alleles to provide tolerance or resistance to pests, diseases, and abiotic stresses [8]. Farmers, instead, use these cultivars for the production of traditional dishes and foods or for trade at niche local markets [9].

To understand the potential of germplasm collection, the genetic and morphological characterizations are usually the most applied and promising methods. Genetic characterization of maize landraces has been performed worldwide in numerous studies, using molecular markers [10,11,12,13,14,15,16,17,18,19,20,21,22,23,24]. Concerning Italian landraces, even if all 562 accessions were morphologically characterized for 17 traits [2,5], only few of them from northern Italy have been investigated at genetic level [7,9,25,26,27,28,29,30].

The present study focuses on the morphological and genetic characterization of thirteen Italian accessions collected in 1954 from the western part of the Emilia-Romagna region.

## 2. Results and Discussion

### 2.1. Morphological Characterization

Morphological characterization of maize accessions was performed according to the UPOV protocol CPVO/TP2/3. For each accession, 34 phenotypic traits were examined, relating to leaf, tassel, ear, and whole plant characteristics. Morphological descriptors and ear figures for each accession are reported in Supplementary Material S1.

The average plant height recorded in these accessions varied from 195 cm for Va221 and Va225 to 253 cm for Va230 (Table 1). Va225 “Nano precoce” was classified as “Early Dwarf Flints” [2,5], characterized by reduced plant (<150 cm) and ear (<14 cm) size and an extreme earliness, whereas the accession analyzed in this study appeared taller than expected. Accessions showed ear insertion heights ranging from 67 cm in Va220W to 114 cm for Va229, with a very small (<40%) ear/plant insertion rate for Va220W, Va222, Va228, Va230, and small (40–45%) for other accessions, whereas Va229 was the only one with an ear/plant insertion rate of 51–55%. Ears were very short in length (<15 cm) forVa221, Va223, and Va224, short (15–18 cm) for Va219, Va220W, Va222, Va225, Va226, Va227, Va228, Va230, and Va231, and long (22–24 cm) for Va229. Ear diameter was very thin (<35 mm) in Va220W, thin (35–40 mm) for Va231, medium (41–45 mm) for Va219, Va221, Va223, Va224, Va226, Va228, and Va230, and large (46–50 mm) for Va222, Va225, Va227, and Va229. All accessions showed a slightly conical ear shape, even though some accessions displayed sometimes conical-shaped ears, like Va219, Va224, and Va227, or a more cylindrical shape for Va225. Most of the accessions had kernel row numbers ranging from 8 to 16, while Va230 ears presented 22 rows. Despite Va228 being traditionally named “Ottofile” (eight-rowed), ears always had a higher number of rows (10–12) and even Brandolini and Brandolini [2,5] classified this accession as “Conical flints and derived races” rather than “Eight-rowed flints”. This is probably due to a continuous cross-contamination with other cultivars before the sampling of 1954, as well as the empirical selection towards ear size. It is believed that the cultivation of “Eight-rowed” agroecotype nearby other racial complexes (conical flints) led to the formation of eight-rowed derived races [2,5], such as 10–12-rowed derived flints, “Cannellino” and “Granturchella” agroecotypes, of which, respectively, Va219, Va226, and Va229 are part. A separate case concerns, instead, Va231, with 8–12-rowed ears, which was classified in the eight-rowed pure group [2]. Kernel type was flint for Va220W, Va223, and Va224, and flint-like for the remaining accessions. Kernel color was generally yellow or yellow-orange, while the Va220W kernels were white; sometimes Va221, Va222, and Va230 exhibited blue-black or black kernels. All accessions generally exhibited white cobs, even if sometimes the cobs of Va225 and Va226 tended to be red. It is reported that traditional Italian maize germplasm is characterized by white cob [31], while the presence of red glumes is associated with recently introduced materials (from the beginning of the XXth century). Anthocyanin coloration is absent in silks for Va222, Va223, Va228, and Va230, and weak for other accessions.

**Figure 1:**
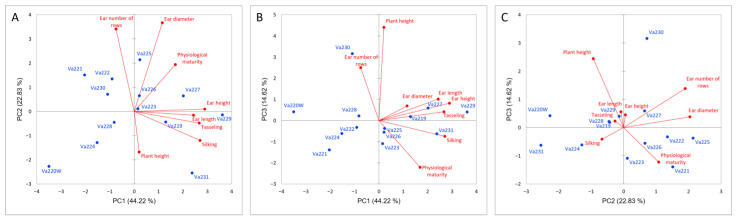
PCA analysis based on morphological measurements of the studied accessions, PC1 vs. PC2 (**A**), PC1 vs. PC3 (**B**), PC2 vs. PC3 (**C**). Maize accessions are reported in blue while directions of morphological vectors are reported in red.

Relationships between morphological traits were investigated using PCA (Figure 1). The first three principal components (PCs) accounted for 81.67% of the total variance. In the first PC, which explained 44.22% of the total variance, the main morphological contributors were ear height (24.55%), tasseling (21.25%), and silking (21.82%). In the second PC (22.83%), ear diameter (40.30%) and kernel row number (34.88%) were the most relevant traits, while for the third PC (14.61%), the main contributor was plant height (58.09%).

**Figure 2:**
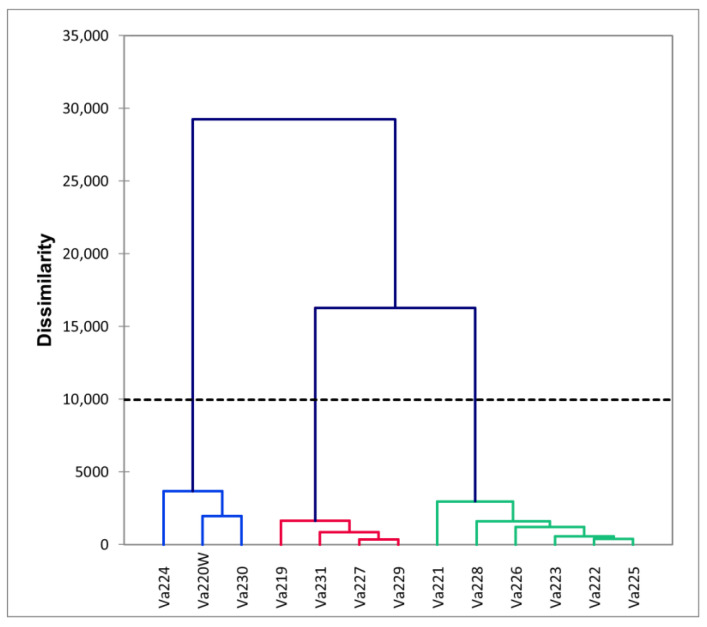
Dendrogram derived from morphological measurements, different colors highlight the clusters of accessions that are morphologically similar.

The dendrogram, reported in Figure 2, identified three different clusters based on phenotypic differences (Figure S1). Comparing the dendrogram with the PCA (Figure 1A), it is possible to speculate that the first cluster is characterized by early flowering and maturing accessions (Va220W, Va224, and Va230), which were also characterized by short ears, with a small insertion on the stalk (Figure S1). The second group is located on the PCA plot in the direction of increasing vectors for ear height, ear length, tasseling, and silking (Figure 1A); this group is characterized by accessions of late flowering and maturity. The ears were higher on the stalk and ear length was longer that those of accessions in clusters 1 and 3 (Figure S1). The remaining accessions are in the above half of the plot, with the only exceptions of Va228, formerly named “Ottofile” (eight-rowed), but with 12 kernel rows, and Va231, another “Ottofile” cultivars, whose ear phenotype is more typical of the accession name. The accessions in this third cluster have intermediate flowering and late maturity (Figure S1).

The collection under investigation presented interesting morphologic variability. Flowering traits are related to the different growing areas; many accessions derive from hills and mountains where the favorable season for maize growing is limited; short-cycle maize varieties were also preferred in the plains because of scarcity of water for irrigation or suitability for use as a second crop after wheat [2]; maize landraces with names such as “Quarantino” or “Cinquantino” reflect such characteristics, as well as landraces with names such as “Agostano”, meaning that the landrace was ready to be harvested in August. Traditionally, maize cultivars characterized by ears with big diameter were preferred because the presence of a big inner cob was associated with an increased resistance or tolerance to summer drought; the big cob diameter and conical shape of the ear are characteristics of the “Conical Flints” racial complex, according to Brandolini and Brandolini [5]. Plant height and ear height, which are very uniform in modern cultivars, are very variable within each accession of traditional cultivars. Short plants with ears inserted at a reduced height may benefit from lodging resistance, and plant height is positively correlated with plant cycle length.

### 2.2. Genetic Characterization

The genetic characterization of the thirteen accessions was performed using ten SSR markers, which are still considered some of the best choices to analyze genetic diversity within and among populations and are widely applied for Italian maize landraces [9,25,30].

Descriptive statistics over all SSR loci and accessions were investigated using the allele dataset of all 820 analyzed maize individuals (Table S1). Summary statistics calculated for both loci and populations are provided in Table 2.

A mean of 7.4 different alleles (N) was recorded over all loci, ranging from four (*umc1401*) to ten alleles (*umc1075* and *umc1786*), for a total of 74 different alleles. Examining populations, the number of alleles varied from 30 for Va227 to 54 for both Va222 and Va223, with a mean value of 41.54 alleles. The numbers of different alleles detected in these accessions are similar to those detected in different Emilia-Romagna maize accessions previously analyzed [28,29] and in other studies conducted worldwide [11,19,20,30]. Most loci were polymorphic for all populations, except for *umc1401*, *umc1786*, and *phi076* in Va226, Va227, and Va228, respectively. The presence of some monomorphic loci is common in various studies [9,13,20,27,28,29,30] and may be the consequence of the selection by local farmers for specific characters. The high number of different alleles and level of polymorphism observed suggest a high genetic variability within populations, compatible with the allogamous nature of maize and the expected multi-genotype constitution of landraces. Fourteen private alleles were detected among all accessions: one in Va220W (*phi076*), Va224 (*phi031*), Va225 (*phi084*), and Va227 (*phi127*), two in Va222 (*umc1327*), three in Va226 (*phi076* (2) and *phi084*), and five in Va223 (*phi127*, *phi076*, *umc1941* (2), and *umc1786*) (Table 3). All the observed private alleles were present at frequencies lower than 5%, except for marker *phi084* (7.63%) in Va225, and thus they cannot be used as food traceability tools, as suggested by Palumbo et al. [9].

The number of observed alleles (N_a_) varied from 2.77 for both *phi084* and *umc1401* to 6.00 for *umc1075*, with a mean value of 4.15, and from 3.00 in Va227 to 5.40 in Va222 and Va223 across populations. The number of expected alleles (N_e_), always lower than N_a_, ranged from 1.80 for *phi084* to 3.23 for *umc1075* and, across populations, from 2.05 in Va224 to 2.74 in Va222, with a mean value of 2.30. The N_a_ values recorded in this work were higher, considering other accessions from the same region previously examined by Stagnati et al. [28,29], confirming the presence of good genetic diversity in the collection. The Shannon information index (I), used to characterize population diversity, had a mean value of 0.93 over all loci and populations, consistent with previous works [9,27,29,30]. The mean value of PIC over all loci was 0.54, with a minimum of 0.41 (*phi084*) and a maximum of 0.71 (*umc1075*), providing an estimation of the ability of each locus to discriminate among different genotypes [9]. The mean value of PIC over all loci investigated in this work was similar with other studies [9,18,20,28,29]. The average values of both observed (H_o_) and unbiased expected (uH_e_) heterozygosity across all loci and populations were 0.52. Considering the fixation index (F) across markers, five loci may be considered in a condition of Hardy–Weinberg equilibrium, while three loci (*phi084*, *umc1327*, and *umc1941*) showed an excess of heterozygosity (−0.12, −0.19, and −0.13, respectively) and two loci (*phi031* and *umc1786*) showed a lack of heterozygosity (0.07 and 0.30, respectively). Concerning populations, Va219 and Va220W showed a lack of heterozygosity (0.08 and 0.14, respectively), whereas Va224, Va226, and Va227 showed an excess of heterozygosity (−0.08, −0.11 and −0.07, respectively). Wright’s inbreeding coefficient (F_IS_) within individuals measures the reduction in heterozygosity due to non-random mating within each population [32]. In this study, the trend observed is very similar to that for F, for both loci and populations; a similar trend was found in previous studies [9,16,27,28,29]. F_IT_ for each locus was always positive, with a mean value of 0.12, except for *umc1327*, with a value of −0.07. Four loci, *phi031*, *p-bnlg176*, *umc1401*, and *umc1786*, showed a high F_IT_ value (0.19, 0.15, 0.23, and 0.40, respectively), revealing a reduction in heterozygosity for those loci. The F_IT_ values found in this work were generally lower than values obtained in other studies with the same [29,30] and different loci [9,11,12,16,18]. The F_ST_ mean value was 0.13, suggesting that these accessions are characterized by a reduced level of genetic variation among populations for the examined loci, lower than that found in the previous findings [16,18,27,28,29,30]. Maize is an allogamous species that benefits from heterozygosity. Moreover, in germplasm collections, landraces are maintained through random intermating systems to guarantee heterozygosity; the presence of excess heterozygosity means an outbred condition of individuals, which is consistent with landrace maintenance. Sometimes, cases of inbreeding or fixation at some loci may be present due to past events of reduction in genetic variability as a consequence of selection that led to the fixation of some traits of interest to farmers or adaptation to environmental conditions. Nm provides an estimation of gene flow, which includes all mechanisms involved in the movement of genes from one population to another. Values of Nm < 1 were considered as reduced gene flow, suggesting gene isolation among the analyzed populations, according to Grassi et al. [33]. In all 10 SSR loci investigated, Nm values were always higher than 1, ranging from 1.07 (*umc1401*) to 2.55 for *phi127*. The highest values of Nm were recorded for loci *phi127*, *umc1075*, and *phi076* (2.55, 2.41, and 2.04, respectively). Allogamous species and open-pollinated varieties generally have gene flow values that are particularly high [34], but considering landraces conservation, lower Nm values were expected. On average, the Nm values for the germplasm analyzed in this study were higher than those for other accessions from the same region previously examined by Stagnati et al. [28,29], as well as other Italian accessions [27,30]. It is possible that before the sampling of 1954, some landraces were subjected to cross-pollination due to growing proximity, as exemplified by landraces sampled in the same area of Cerignale-Ottone-Bobbio in the province of Piacenza.

### 2.3. PCoA Analysis

Principal coordinates analysis (PCoA), performed starting from a genetic distance matrix of all samples, did not separate individuals into well-defined groups (Figure S2). The first two coordinates explained only 8.71% and 8.05% of the total genotypic variability, of which most remained unexplained. Even though the picture was quite unclear, the first two coordinates clearly separated Va219 and Va227 from the remaining collection, whereas the second coordinate separated Va228 from Va220W, Va221, Va223, and Va225, despite the presence of a few samples in the middle. Except for these three accessions (Va219, Va227, and Va228), the remaining accessions clustered together into a single group, since the high overlapping among them made it difficult to achieve a clear separation. Furthermore, there was no clear correlation between the geographic distribution of those accessions in the three sampling provinces and the findings from PCoA. The low resolution of PCoA can be explained by considering the high intra-population genetic variability and past events of intercrossing between different materials, as suggested by previous studies and Nm statistics [2,35].

**Figure 3:**
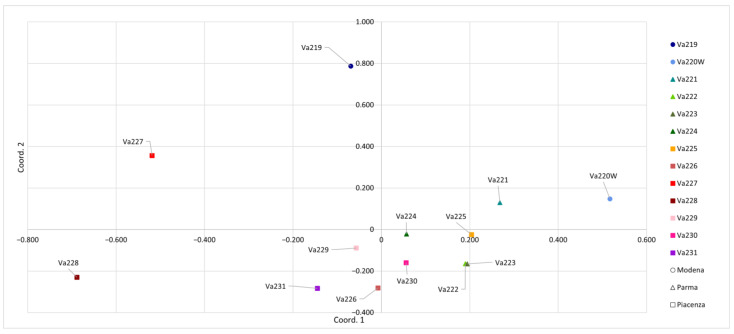
Principal coordinates analysis (PCoA) conducted according to the thirteen accessions.

When PCoA was carried out considering a genetic distance matrix between populations, the separation among accessions became clearer (Figure 3). The first principal coordinate accounted for 22.47% of the total genetic variation and better separated accessions previously clustered together, whereas the second principal coordinate accounted for 19.83% of the total variation. This allowed the good separation of Va219, Va220W, Va227, and Va228 from the other accessions. Moreover, PCoA of populations allowed a better separation between provinces, where two accessions from Modena (Va219 and Va220W) were well separated from the others, while four from Parma (Va221, Va222, Va223, and Va224) clustered together. Va222 and Va223 from Parma overlapped. This result was not expected, since they have different names and were cultivated and sampled in different locations: the first one in the Parma Apennines (Albareto) and the second in Salsomaggiore, in the first hills closed to the Po valley. In traditional germplasms, cases of homonymy and synonymy are particularly abundant and have been reported for maize [27] and other crops [36,37]. Va227 and Va228 were genetically distant, even if they were cultivated and sampled in the same location and classified into the same agroecotype by Brandolini and Brandolini [2,5]. This result may indicate proper maintenance of populations before sampling in 1954. It is possible that common morphological features are a case of convergent evolution or preferential selection by farmers. Findings from PCoA analysis were partially confirmed by pairwise population F_ST_ values: the highest pairwise F_ST_ values were shown between Va220W and Va228 (0.25), Va220 and Va227 (0.24), Va219 and Va228 (0.22), Va227 and Va231 (0.21), Va221 and Va228 (0.20), Va219 and Va226 (0.20), with a mean value of 0.13 (Table S2). The pairwise F_ST_ value for Va222 and Va223 (0.08) was one of the lowest and partially matched the PCoA results.

In support of the low levels of genetic variation explained by PCoA, AMOVA found that among all the variability observed, only 13% (*p* < 0.001) was due to differences among populations, whereas the remaining variation was found within accessions. Other studies reported that maize landraces cultivated at long distances from one another displayed higher levels of differentiation than materials sampled in close proximity [9,16,27,28,29,30].

### 2.4. Genetic Clustering

**Figure 4:**
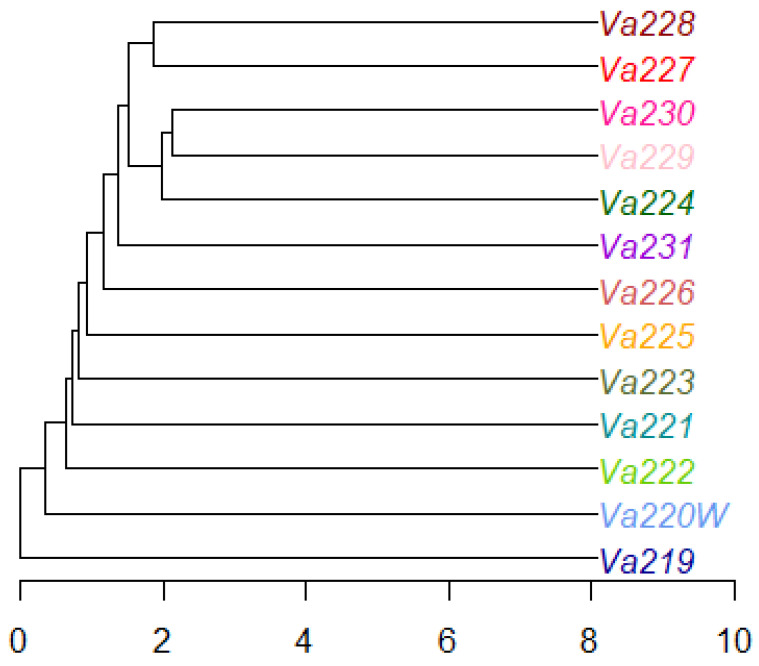
Dendrogram of the thirteen accessions, computed using the UPGMA method.

The radial tree of individuals, computed using the UPGMA method, revealed that only half of all samples clustered well according to their population identification, while the remaining individuals tended to mix with each other (Figure S3). The radial UPGMA tree showed a good grouping of individuals in Va219, Va220W, Va225, Va227, Va228, and Va231, in agreement with the findings of the PCoA. Starting from a matrix of genetic distance between populations, the UPGMA dendrogram was computed, showing that eight out of 13 accessions did not cluster together, while five accessions grouped in one cluster, formed by Va227–Va228 and Va224–Va229–Va230 subclusters (Figure 4). Observing the UPGMA dendrogram, it is possible to notice a correlation between the reduction of genetic distance and the spatial distribution of populations, as well as with PCoA (Figure 3), starting from those of Modena (Va219 and Va220W), followed by Parma (Va221, Va222, and Va223, except for Va224, grouped with Va229 and Va230), and then those of Piacenza (from Va225 to Va231). Interestingly, Va227 and Va228, clustering together, were sampled in the same municipality and were classified in the same agroecotype by Brandolini and Brandolini [2]; Va229 and Va230 were sampled at about 30 km of distance and clustered with Va224, sampled far away, in a different area. It is possible that, despite the sampling distance and traditional classification (Table 4), these materials may have some relationship due to seed exchange between farmers and families. The name “Nostrano”, shared between Va224 and Va230, is used to refer to something “original from this place” and was widely utilized for maize landraces and other crops, though it is not exhaustive in explaining the relationships among Va229, Va230, and Va224.

### 2.5. Analysis of Genetic Structure

Population structure was investigated by STRUCTURE, using the Evanno method to determine the best level of K [38]. The highest ∆K value (∆K = 59.22) was observed at K = 4. Structure analysis revealed that only 296 of 820 samples (36.10%) showed strong ancestry association (>0.9), while 150 individuals showed a membership coefficient (Q) of 0.8 < Q ≤ 0.9, and 95 samples of 0.7 < Q ≤ 0.8 in any of the determined clusters. A total of 279 individuals (34.02%) among all accessions analyzed were considered admixed, with a Q < 0.7 [9,39]. According to accessions, no accession showed strong ancestry association, two accessions showed means of 0.8 < Q ≤ 0.9 and four accessions showed means of 0.7 < Q ≤ 0.8, while seven of 13 accessions remain admixed (Table S3).

**Figure 5:**
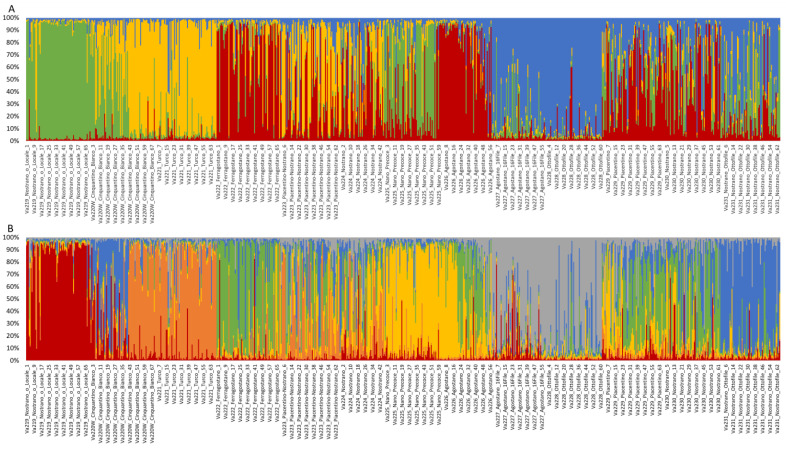
Population genetic structure of the thirteen maize accessions, as estimated by STRUCTURE, at K = 4 (**A**) and K = 6 (**B**). Different colors correspond to different ancestral populations.

Va219 was clearly assigned to a cluster with a membership mean of 0.887, as was Va228, with a Q of 0.894 (Figure 5A). Va222 and Va226 were grouped in the same cluster, with a membership coefficient of 0.716 and 0.779, respectively, while Va221 and Va227 were assigned to two different clusters, with Q = 0.787 and Q = 0.771, respectively. The remaining accessions, Va223, Va224, Va225, Va229, Va230, and Va231, were considered admixed, with only 122 of 368 samples associated with a specific cluster (Q > 0.8).

Structure analysis, at K = 6, revealed an alternative level of population structure (Figure 5B and Table S4). As observed for K = 4, Va219 and Va221 clustered separately, and Va227 and Va228 clustered together. At K = 6, Va225 and Va231 were assigned to two clusters, while Va222 and Va226 became admixed. Va223, Va224, Va229, and Va230 were still considered admixed populations.

Comparing the UPGMA dendrogram to population structure analysis, it is also possible to notice that at least one K, K = 4 or K = 6, all ungrouped populations of the tree, except for Va220W and Va223, were assigned to a specific cluster by STRUCTURE, whereas Va224, Va229, and Va230 were always plotted as admixed populations at both K levels.

The grouping of Va227 and Va228 in the same cluster agrees with the UPGMA dendrogram, thus suggesting that these accessions derive from the same ancestral population, even if the PCoA separates the two accessions. According to the classification by Brandolini and Brandolini [2], these accessions are related, both classified in the same “Montano” agroecotype and cultivated in the same location. The high membership coefficients of Va219 and Va226 to respective clusters were not expected, since accessions of the “10–12 rowed-derived flints” agroecotype, of which Va219 is part, and the “Cannellino” agroecotype for Va226 were considered the result of contamination of “Eight-rowed flint” landraces with other cultivars; therefore, an admixed population was expected. Structure analysis results for Va219 were in agreement with the PCoA analysis and the UPGMA dendrogram and may confirm the fact that Va219 was sampled far away from the other accessions, or that it derives from ancestral populations that are not part of the present collection. Another unusual case is represented by Va221 and Va222 since, despite belonging to the same agroecotype, they originated from two distinct ancestral populations; this is a possible example of convergent evolution due to farmers’ selection for desired traits. Moreover, according to PCoA and F_ST_, Va222 and Va223 were expected to be more related. Va225 is the only accession belonging to the “Early Dwarf Flints” genetic complex but is considered an admixed population, as expected, since morphological characterization showed a lack of distinctive traits specific to that racial complex.

The presence of many admixed genotypes and the relatively low pairwise F_ST_ values recorded between populations may be explained by the fact that some events of gene flow or pollen exchange probably occurred before the sampling of 1954, since all landraces were cultivated and maintained as open-pollinated populations by farmers [40]. In the case of panmictic reproduction and possible seed exchange between farmers, while they applied continuous selection of few desired traits, genetic distinctiveness was probably missed, even if landraces were clearly different at morphological level [21]. This behavior may explain the fact that some landraces with unique morphological traits or belonging to unique racial complexes, such as Va220W (the only accession with white kernels analyzed in this study) or Va225 (the only member of the “Early Dwarf Flints” complex), were considered admixed populations. When considering the names of the accessions, it can be noticed that some of them share the same name, such as Va223 and Va229, both named “Piacentino”, or names like “Nostrano”, or names indicative of the vegetative cycle (“Agostano” and “Ferragostano”), which are very common in local cultivars. According to the genetic analysis, it is possible to speculate that Va229 and Va230 were the only variants of the same landrace, even if the morphological clusterization placed these accessions very far apart (Figure 1 and Figure 2), while in other cases (i.e., Va219), the same name has been used for different materials.

When comparing the results with the PCA and dendrogram of morphological traits, a limited correspondence has been noted. Clear relationships between morphology and genetics have not been evidenced. It is probable that morphological analysis is able to distinguish accessions of admixed origin, which are more morphologically distinguishable than from a genetic point of view. In this regard, limited correspondence was observed for Va220W, which is one of the most different accessions, resulting from PCA and genetic clustering.

## 3. Materials and Methods

### 3.1. Germplasm and Field Management

Thirteen maize landraces conserved ex situ in the germplasm collection at CREA-CI in Bergamo were analyzed in this work. The collection sites were located in three provinces of the western Emilia-Romagna region, Modena, Parma, and Piacenza, with 2, 4, and 7 accessions, respectively. The accessions were as follows: Va219 “Nostrano o Locale”, Va220W “Cinquantino Bianco”, Va221 “Turco”, Va222 “Ferragostano”, Va223 “Piacentino o Nostrano”, Va224 “Nostrano”, Va225 “Nano Precoce”, Va226 “Agostano”, Va227 “Agostano 16 file”, Va228 “Ottofile”, Va229 “Piacentino”, Va230 “Nostrano”, and Va231 “Nostrano Ottofile”. All these accessions were part of a wider restoration project called RICOLMA, during which various traditional maize cultivars from the Emilia-Romagna region were characterized. Some of them were analyzed in two previous studies [28,29]. More detailed information on the thirteen germplasm sources is reported in Table 4.

The field trial prepared for accession characterization was located at CREI-CERZOO (45°0.303960′ N, 9°42.252360′ E, San Bonico, Piacenza, Italy) and sown on 27 April 2018. Each accession plot consisted of 5 rows 5 m long, spaced 80 cm apart from each row, and 1 m aisle on the hedge; for each row, 20 seeds were planted. The field trial was managed according to appropriate agricultural practices for maize nursery cultivation. Morphological characterization was performed on the entire plot, relying on the UPOV protocol CPVO/TP2/3, examining 34 phenotypic traits. Tasseling, silking, and physiological maturity were collected when 50% of plants in each plot reached phenological stage, expressed as days after sowing, and then converted to growing degree days (GDD) with the formula:

$$GDD=\sum_{i}^{n}\left[\frac{Tmin+Tmax}{2}-10\right]$$

where *n* is the day of tasseling, silking, or physiological maturity, *i* is the sowing day, and *Tmin* and *Tmax* are the minimum and maximum daily temperatures. All daily temperatures below 10 °C or over 30 °C were substituted by the cardinal temperature for maize growing (10 and 30 °C, respectively) [41]. Maize accessions were reproduced by manual random-intermating, avoiding self-pollination. Seed stocks are stored in the germplasm bank of the Department of Sustainable Crop Production at Università Cattolica del Sacro Cuore, Piacenza (Italy) and the Department of Earth and Environmental Sciences, Università degli Studi di Pavia, Pavia (Italy).

### 3.2. DNA Extraction and PCR Amplification

Leaf tissues were collected from all plants at the 5th leaf stage. Genomic DNA was extracted from 10 g of lyophilized tissues according to the “96-Well Plate Plant Genomic DNA Miniprep Kit” (BIO BASIC Europe s.r.l., Milano, Italy), following the manufacturer’s instructions with minor modifications, as previously reported by Stagnati et al. [27]. Genomic DNA was visualized using 1% agarose gel electrophoresis containing Midori Green stain (Nippon Genetics Europe, Düren, Germany). The quantity and quality of the extracted DNA were evaluated with the NanoPhotometer^®^ NP80 UV-Vis spectrophotometer (Implen GmbH, Munich, Germany). In this study, 820 plants were analyzed: 47 for Va224, 57 for Va226, 59 for both Va225 and Va227, 61 for Va228, 63 for Va230, 65 for Va231, 66 for Va229, 67 for Va222, 68 for both Va220W and Va223, and 70 for Va219 and Va221.

The genetic characterization was performed using 10 SSR markers, as previously reported [27,28,29,30] (Table S5). PCR amplification was performed in 96-well plates using a GeneAmp 2700 thermocycler (Applied Biosystem, ThermoFisher Scientific, Monza, Italy), under the same conditions described in Stagnati et al. [27,28]. PCR products of different fluorescence and size were multiplexed and separated using an ABI 3130xl genetic analyzer sequencer (Applied Biosystems, Waltham, MA, USA), according to the manufacturer’s instructions; GeneScan™ 500 ROX™ was used as the size standard. Visualizations and sizing of the PCR fragments were performed using GeneMapper software version 4.0 (Applied Biosystems).

### 3.3. Statistical Analysis

Morphological measurements were used to compute a principal component analysis and dendrogram based on the dissimilarity matrix of Euclidean distances and Ward’s agglomeration method using the XLSTAT add-on [42] for Microsoft Excel.

Recorded molecular data were analyzed through the GenAlEx 6.5 Excel package [43,44] to compute population statistics, F-statistics, Nei’s unbiased genetic distance, principal coordinates analysis (PCoA), and analysis of molecular variance (AMOVA) according to default parameters. Polymorphic information content (PIC) values were calculated for each SSR with PowerMarker 3.25 software [45].

Detected alleles from each individual were used to compute UPGMA trees using R software [46], considering both all 820 samples as separated individuals and after aggregating them in their accession population. Both UPGMA trees were computed using the unweighted pair group method with the arithmetic mean (UPGMA) method through the *upgma* function available in the *phangorn* R package [47] and then plotted with the *ape* package [48]. The UPGMA tree of individuals was obtained from a genetic distance matrix, calculated by the *meandistance.matrix* function with the *polysat* package [49], whereas the population genetic distance matrix was computed using GenAlEx 6.5.

The genetic structure of the thirteen accessions was established using a Bayesian clustering algorithm implemented in STRUCTURE 2.3.4 software [50]. The “admixture model” and the “correlated allele frequency model” options were selected, as suggested in previous works [9,50]. Ten independent replicate simulations were computed for each level of K, ranging from 2 to 20, with a burn-in of 2 × 10^5^ and 10^6^ Markov Chain Monte Carlo steps. Then, the most likely estimation of K was selected according to the method of Evanno [9,38]. Membership coefficient values were plotted as a histogram using an Excel spreadsheet.

## 4. Conclusions

The genetic characterization of the 13 maize accessions, even though conducted with a limited number of SSR markers, showed high intra-population variability within local cultivars from the west Emilia-Romagna region. The number of alleles, heterozygosity and inbreeding coefficient values were consistent with the allogamous nature of maize. Cluster and structure analysis revealed that four out of thirteen accessions of this germplasm, Va219, Va221, Va227, and Va228, were clearly differentiated from other accessions of admixed origin. In the future, it would be of interest to increase the number of accessions investigated at genetic level by the means of GBS that is able to provide a higher number of markers, allowing a better comprehension of the relationships between different cultivars. Morphological analysis revealed that accessions were phenotypically different, each characterized by peculiar traits. The absence of correspondence between genetic and morphological clusterization, along with the fact that admixed genotypes were phenotypically different, suggest that even in the presence of genetic exchange among different materials, the selection pressure operated by the environment and local farmers played a key role in germplasm differentiation.

Despite the limited usefulness of landraces for direct animal feeding, the presence of high genetic variability allows their employment and cultivation in marginal areas, for the production of traditional dishes and foods, or for niche local markets. Nowadays, maize breeding relies on a limited genetic base, leaving the majority of genetic diversity unexplored and unexploited inside local varieties and unimproved materials. The direct use of landraces and local varieties in breeding programs is difficult because of their low agronomic value; however, pre-breeding work and crosses between landraces and elite lines may be a reasonable compromise to introduce new genetic variation without a high detrimental effect on agronomic performances in order to develop new breeding materials and commercial cultivars. Additionally, local and traditional varieties have a long history of natural selection and adaptation to marginal environments or suboptimal conditions. In the current frame of climate change, it would be of interest to investigate these genotypes for their traits of adaptation by the means of landscape genomics to highlight genomic regions to be selected or introgressed into elite varieties and breeding germplasm. In this way, the plasticity of hybrids towards suboptimal environments will be increased.

## Figures and Tables

**Table 1:** Main morphological descriptors for each accession; mean values and standard deviations are reported.

Accession	Plant Height (cm)	Ear Height (cm)	Ear Length (cm)	Ear Diameter (mm)	Ear Number of Rows	Tasseling (GDD)	Silking (GDD)	Physiological Maturity (GDD)
Va219	225 ± 7.1	94 ± 5.5	16.7 ± 2.1	44 ± 1	12 ± 0	662	707	1414
Va220W	228 ± 14.8	67 ± 13	15 ± 1.4	32.2 ± 2.7	12 ± 1.4	593	632	1317
Va221	195 ± 14.6	78 ± 2.7	13.2 ± 1.1	43.6 ± 5.1	13.6 ± 1.7	604	632	1446
Va222	210 ± 17	74 ± 14.7	15.2 ± 0.8	45.6 ± 3.9	14.4 ± 0.9	618	692	1430
Va223	207 ± 18.6	86 ± 11.9	14.8 ± 2.3	41.8 ± 2.8	12.4 ± 2.6	646	692	1446
Va224	205 ± 5	72 ± 16.8	13.4 ± 0.9	40.8 ± 4.7	11.6 ± 1.7	646	692	1317
Va225	195 ± 14.7	85 ± 20.6	18 ± 1.6	48.2 ± 4.5	14.8 ± 1.1	632	677	1430
Va226	199 ± 14.3	91 ± 7.4	17.4 ± 1.3	45.4 ± 3.1	12.8 ± 2.3	632	677	1398
Va227	227 ± 13	95 ± 11.2	16 ± 1.4	48.25 ± 3.1	13.5 ± 1	677	739	1430
Va228	238 ± 21.7	81 ± 15.2	15 ± 2.5	43 ± 2.2	11.6 ± 0.9	618	662	1430
Va229	220 ± 22.4	114 ± 10.8	21.6 ± 1.7	46 ± 4.1	12 ± 1.4	662	739	1430
Va230	253 ± 12	87 ± 19.2	16 ± 1.4	44.8 ± 5.6	16 ± 4.5	632	646	1349
Va231	226 ± 25.3	98 ± 12.5	18.4 ± 2.4	35.2 ± 2.3	10.4 ± 1.7	662	771	1430

GDD = growing degree days.

**Table 2:** Genetic parameters calculated according to the ten SSRs and thirteen accessions in this study. The following parameters are reported: number of different alleles (N), number of observed (N_a_) and effective (N_e_) alleles, Shannon index (I), polymorphic information content (PIC), observed (H_o_) and unbiased expected (uH_e_) heterozygosity, Wright’s inbreeding coefficient (F, F_IS_, F_IT_, F_ST_), and gene flow (Nm).

	N	N_a_	N_e_	I	PIC	H_o_	uH_e_	F	F_IS_	F_IT_	F_ST_	Nm
*phi127*	7	4.08	2.01	0.86	0.47	0.48	0.49	0.01	0.02	0.11	0.09	2.55
*phi076*	7	3.00	1.83	0.71	0.42	0.43	0.43	−0.01	−0.01	0.10	0.11	2.04
*phi031*	7	4.54	2.51	1.02	0.58	0.50	0.55	0.07	0.09	0.19	0.12	1.91
*umc1075*	10	6.00	3.23	1.34	0.71	0.69	0.69	−0.02	−0.01	0.08	0.09	2.41
*phi084*	6	2.77	1.80	0.68	0.41	0.47	0.42	−0.12	−0.14	0.02	0.13	1.61
*umc1327*	8	4.23	2.16	0.90	0.53	0.62	0.51	−0.19	−0.23	−0.07	0.13	1.70
*p-bnlg176*	6	4.46	2.58	1.07	0.67	0.60	0.59	−0.02	−0.03	0.15	0.17	1.18
*umc1941*	9	4.38	2.44	1.02	0.59	0.64	0.57	−0.13	−0.14	0.01	0.13	1.64
*umc1401*	4	2.77	1.87	0.68	0.46	0.39	0.41	0.03	0.05	0.23	0.19	1.07
*umc1786*	10	5.31	2.53	1.02	0.60	0.37	0.52	0.30	0.28	0.40	0.17	1.18
Mean all loci	7.40	4.15	2.30	0.93	0.54	0.52	0.52	−0.01	−0.01	0.12	0.13	1.73
SE	0.60	0.32	0.17	0.08	0.03	0.05	0.04	0.06	0.04	0.04	0.01	0.16
Va219	40	4.00	2.29	0.91	-	0.49	0.51	0.08	0.02	-	-	-
Va220W	35	3.50	2.15	0.80	-	0.37	0.45	0.14	0.17	-	-	-
Va221	42	4.20	2.33	0.97	-	0.50	0.54	0.05	0.07	-	-	-
Va222	54	5.40	2.74	1.12	-	0.57	0.60	0.04	0.05	-	-	-
Va223	54	5.40	2.65	1.11	-	0.61	0.60	−0.05	−0.03	-	-	-
Va224	39	3.90	2.05	0.87	-	0.53	0.49	−0.08	−0.09	-	-	-
Va225	45	4.50	2.64	1.11	-	0.62	0.61	−0.02	−0.03	-	-	-
Va226	45	4.50	2.41	0.98	-	0.60	0.54	−0.11	−0.11	-	-	-
Va227	30	3.00	2.08	0.75	-	0.45	0.44	−0.04	−0.04	-	-	-
Va228	34	3.40	2.10	0.82	-	0.50	0.46	−0.05	−0.08	-	-	-
Va229	44	4.40	2.10	0.89	-	0.51	0.49	−0.07	−0.04	-	-	-
Va230	41	4.10	2.23	0.93	-	0.53	0.52	−0.03	−0.02	-	-	-
Va231	37	3.70	2.07	0.83	-	0.48	0.48	−0.02	−0.01	-	-	-
Mean all pop	41.54	4.15	2.30	0.93	-	0.52	0.52	−0.01	−0.01	-	-	-
SE	1.89	0.14	0.07	0.03	-	0.02	0.02	0.02	0.02	-	-	-

**Table 3:** List of private alleles detected in the accessions.

Accession	Locus	Allele	Allele Frequency
Va220W	*phi076*	153	0.0074
Va222	*umc1327*	98	0.0076
Va222	*umc1327*	102	0.0076
Va223	*phi127*	102	0.0149
Va223	*phi076*	159	0.0224
Va223	*umc1941*	90	0.0076
Va223	*umc1941*	96	0.0076
Va223	*umc1786*	146	0.0154
Va224	*phi031*	209	0.0333
Va225	*phi084*	146	0.0763
Va226	*phi076*	167	0.0094
Va226	*phi076*	174	0.0283
Va226	*phi084*	156	0.0088
Va227	*phi127*	116	0.0169

**Table 4:** Detailed information about maize germplasm used in this study.

Accession	Denomination	Sampling Location	Racial Complex	Local Race	Agroecotype	Latitude (North)	Longitude (East)	Altitude (m asl)
Va219	Nostrano o Locale	Modena (MO)	Eight-Rowed Flints and Derived Races	10–12 Rowed Derived Flints	10–12 Rowed Derived Flints	44°35′	10°50′	134
Va220W	Cinquantino bianco	Modena (MO)	Conical Flints and Derived Races	Biancone	Quarantino Bianco	44°35′	10°54′	84
Va221	Turco	Borgo Val di Taro (PR)	Conical Flints and Derived Races	Poliranghi	Maggengo	44°30′	09°45′	411
Va222	Ferragostano	Albareto (PR)	Conical Flints and Derived Races	Poliranghi	Maggengo	44°27′	09°43′	512
Va223	Piacentino o Nostrano	Salsomaggiore (PR)	Conical Flints and Derived Races	Barbina	Barbina	44°49′	09°59′	157
Va224	Nostrano	Talignano di Sala (PR)	Conical Flints and Derived Races	Barbina	Barbina	44°45′	10°14′	182
Va225	Nano precoce	Ottone (PC)	Early dwarf flints	Trenodi	Nano	44°38′	09°20′	800
Va226	Agostano	Cerignale (PC)	Eight-Rowed Flints and Derived Races	Cannellino	Cannellino	44°42′	09°21′	600
Va227	Agostano 16 file	Bobbio (PC)	Conical Flints and Derived Races	Montano	Montano	44°46′	09°23′	700
Va228	Ottofile	Bobbio (PC)	Conical Flints and Derived Races	Montano	Montano	44°46′	09°23′	800
Va229	Piacentino	Bobbio (PC)	Eight-Rowed Flints and Derived Races	Cannellino	Granturchella	44°46′	09°23′	700
Va230	Nostrano	Gramizzola-Ottone (PC)	Conical Flints and Derived Races	Montano	Costarolo	44°37′	09°21′	645
Va231	Nostrano ottofile	Cerignale-Bobbio (PC)	Eight-Rowed Flints and Derived Races	Ottofile tardivo	Ottofile tardivo	44°46′	09°23′	437

## Data Availability

All relevant data are contained within the article or Supplementary Materials.

## Supplementary Materials

The following supporting information can be downloaded at: https://www.mdpi.com/article/10.3390/plants13071030/s1, Figure S1: Boxplot of phenotypic traits according to dendrogram clustering; Figure S2: Principal coordinates analysis (PCoA) of 820 samples characterized by 10 SSR markers; Figure S3: Radial UPGMA tree of 820 individuals of the thirteen maize accessions; Material S1: Morphological descriptors and ear figures for each accession; Table S1: Allele dataset of all 820 maize individuals analyzed in this study; Table S2: Pairwise population FST values; Table S3: Proportion of membership of each accession at K = 4; Table S4: Proportion of membership of each accession at K = 6; Table S5: Detailed information about primer pairs used in this study. For each microsatellite locus, we report the following details: marker name, locus name, forward and reverse primer sequences, chromosomal location (Bin), annealing temperature (Ta), SSR motif, and amplicon size in bp.
