# Supplementary figures and images for: Morphological and Genetic Characterization of Maize Landraces Adapted to Marginal Hills in North-West Italy
Source: Plants (Basel). 2024 Apr 5;13(7):1030. doi: 10.3390/plants13071030 (PMC11013090; doi:10.3390/plants13071030)

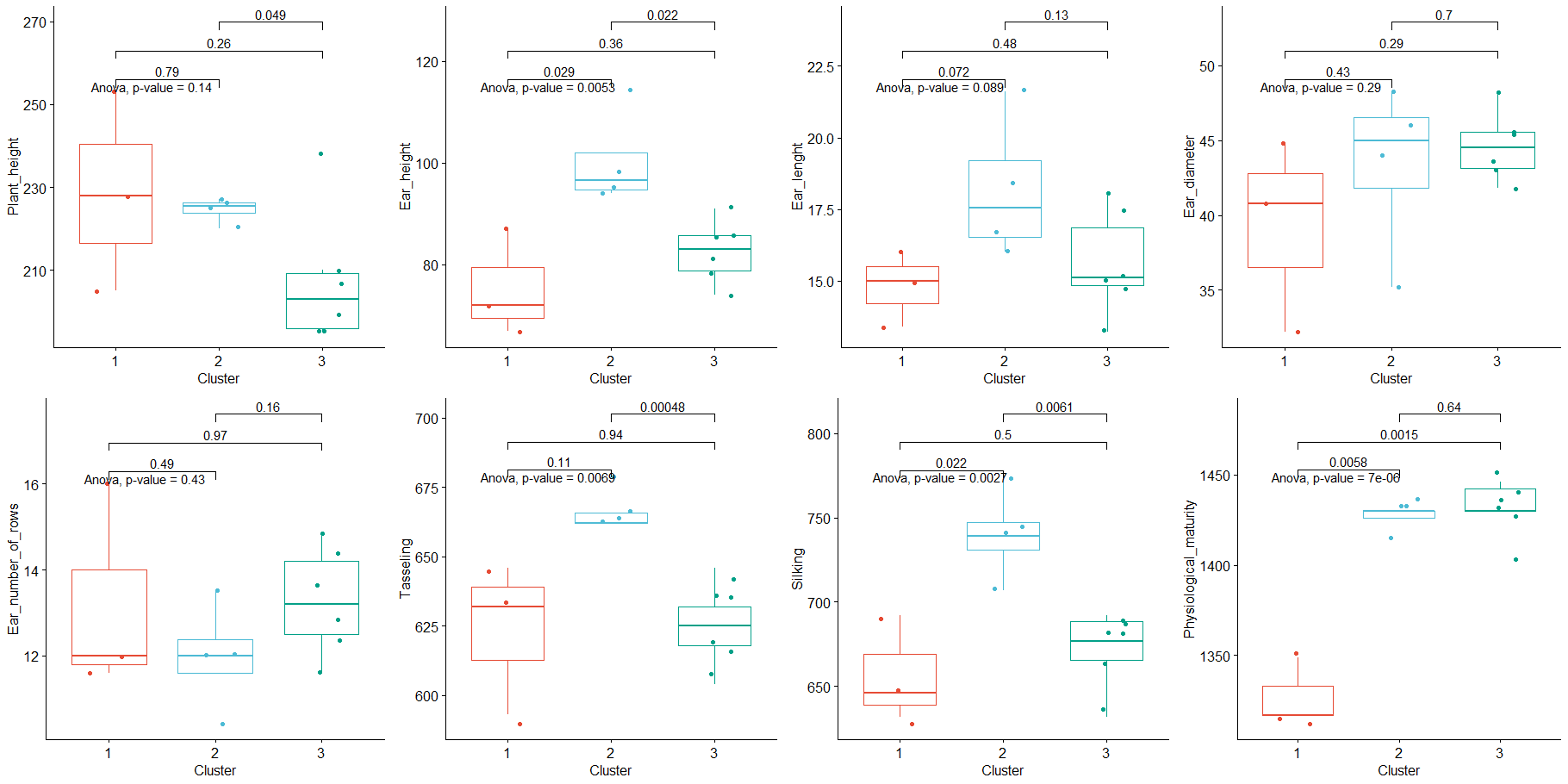

Supplement: Supplementary file 1 [file plants-13-01030-s001.zip › Di Pasquale et al_Supplementary Figure S1.tif]

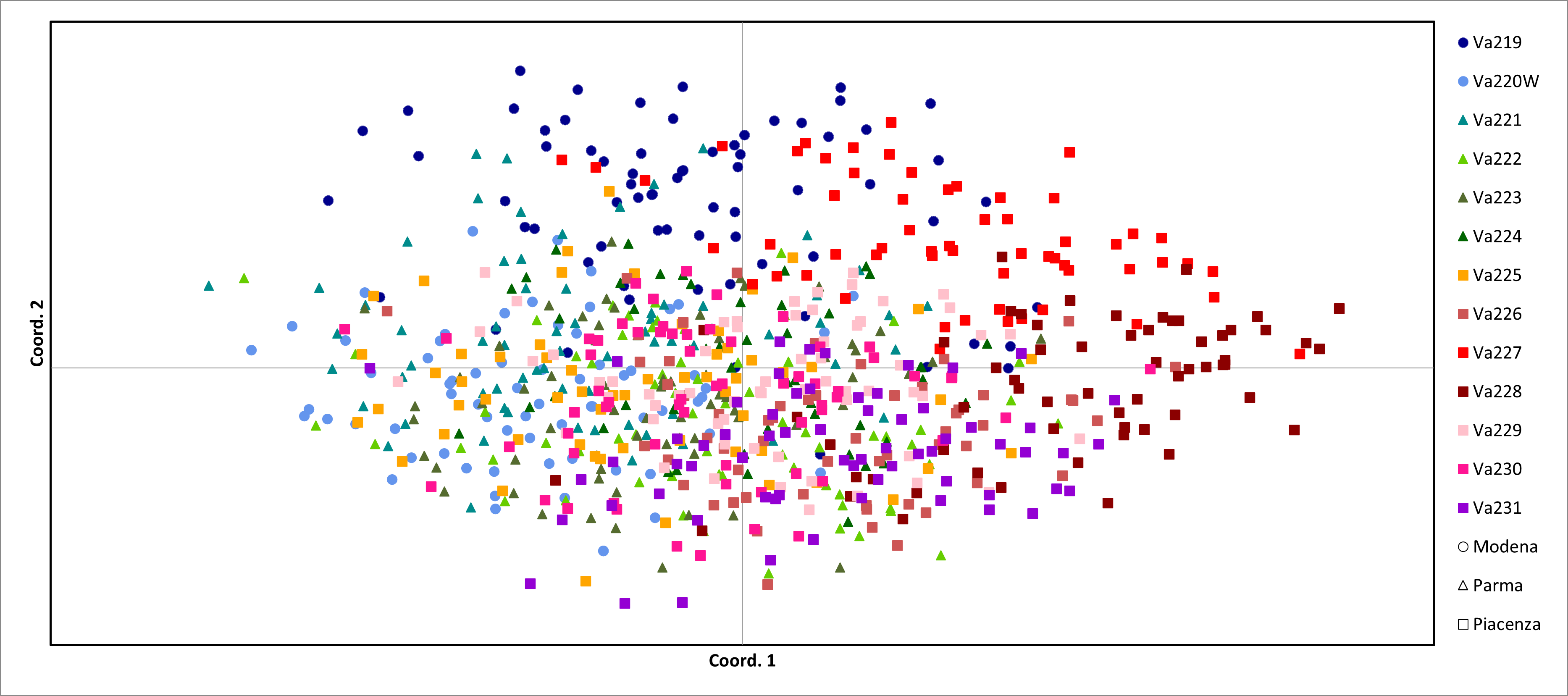

Supplement: Supplementary file 1 [file plants-13-01030-s001.zip › Di Pasquale et al_Supplementary Figure S2.tif]

- Va219
- Va220W
- Va221
- Va222
- Va223
- Va224
- Va225
- Va226
- Va227
- Va228
- Va229
- Va230
- Va231

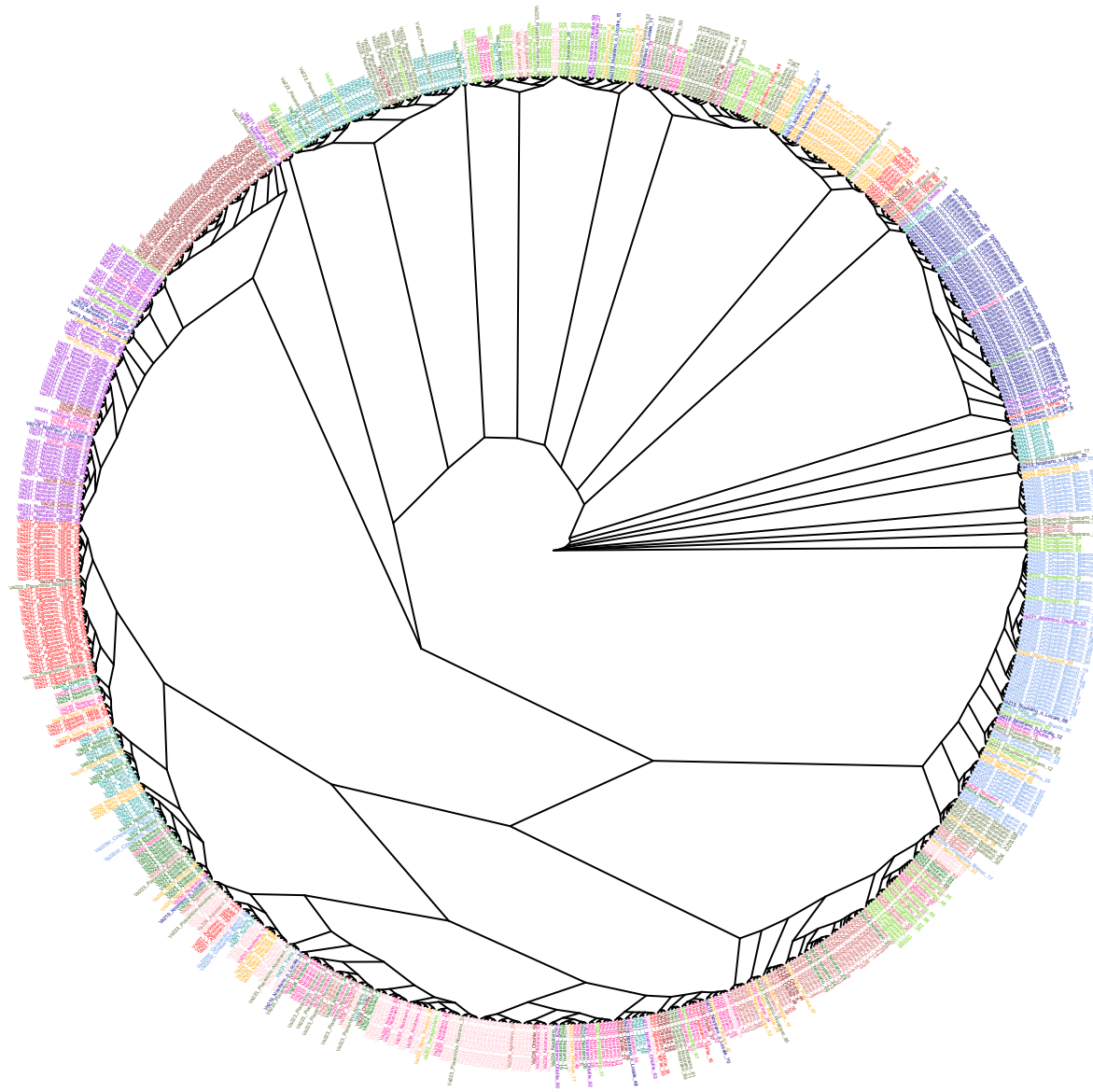

Supplement: Supplementary file 1 [file plants-13-01030-s001.zip › Di Pasquale et al_Supplementary Figure S3.pdf]
